# “WHERE THERE IS A WILL, THERE IS A WAY”: STRENGTH AND RESILIENCE IN THE STORIES OF SOUTH ASIAN OLDER ADULTS

**DOI:** 10.1093/geroni/igae098.1730

**Published:** 2024-12-31

**Authors:** Mushira Khan

**Affiliations:** Mather Institute, Evanston, Illinois, United States

## Abstract

Productive aging is deeply connected to the realms of communal and associational life (Russell, 2011). Asset-based approaches facilitate productive aging by harnessing existing gifts and strengths within individuals and their communities and fostering a sense of purpose in older adults (Hornby-Turner et al., 2017). Despite this evidence, little is known about the specific experiences of South Asian American (SAA) older adults in relation to well-being. To address this gap, this qualitative study explored the strengths that contribute to their well-being through in-depth interviews with 32 SAA adults (44% female; mean age=58.3 years) ages 50+. Each interview lasted between 30 and 90 minutes and was audio-recorded with the participant’s consent. Thematic analysis of the interview data revealed that several key assets facilitated a sense of purpose in SAA older adults, including robust local and global social support networks, often maintained through social media platforms such as WhatsApp and participation in cultural events, strong affectual ties between generations, self-transcendence through meditation and mindfulness-based practices, a sense of resilience, and creative coping strategies, particularly among late-life immigrants. These findings suggest that an asset-based approach can empower and support the well-being of SAA older adults by recognizing and leveraging their existing strengths, resources, and personal, familial, and cultural assets. For example, enhancing digital literacy in this population may offset loneliness through more effective engagement with digital social activities. Overall, policymakers, practitioners, and researchers may want to capitalize on these strengths when developing interventions or programs aimed at promoting productive aging among SAA older adults.

